# Reflection as a Deliberative and Distributed Practice: Assessing Neuro-Enhancement Technologies via Mutual Learning Exercises (MLEs)

**DOI:** 10.1007/s11569-017-0287-4

**Published:** 2017-03-24

**Authors:** Hub Zwart, Jonna Brenninkmeijer, Peter Eduard, Lotte Krabbenborg, Sheena Laursen, Gema Revuelta, Winnie Toonders

**Affiliations:** 1 Institute for Science, Innovation and Society, Department of Philosophy and Science Studies, Radboud University, P.O. Box 9010, 6500 GL Nijmegen, the Netherlands; 2 Experimentarium, Copenhagen, Denmark; 3 Universitat Pompeu Fabra, Barcelona, Spain

**Keywords:** Emerging technologies, Responsible research and innovation, Neuro-enhancement, Mutual learning exercises, Upstream public engagement, Moral deliberation

## Abstract

In 1968, Jürgen Habermas claimed that, in an advanced technological society, the emancipatory force of knowledge can only be regained by actively recovering the ‘forgotten experience of reflection’. In this article, we argue that, in the contemporary situation, critical reflection requires a deliberative ambiance, a process of mutual learning, a consciously organised process of deliberative and distributed reflection. And this especially applies, we argue, to critical reflection concerning a specific subset of technologies which are actually oriented towards optimising human cognition (neuro-enhancement). In order to create a deliberative ambiance, fostering critical upstream reflection on emerging technologies, we developed (in the context of a European 7^th^ Framework Programme project on neuro-enhancement and responsible research and innovation, called NERRI) the concept of a mutual learning exercise (MLE). Building on a number of case studies, we analyse what an MLE involves, both practically and conceptually, focussing on key aspects such as ambiance and expertise, the role of ‘genres of the imagination’ and the profiles of various ‘subcultures of debate’. Ideally, an MLE becomes a contemporary version of the Socratic agora, providing a stage where multiple and sometimes unexpected voices and perspectives mutually challenge each other, in order to strength-en the societal robustness and responsiveness of emerg-ing technologies.

## Introduction

Almost half a century ago, the German philosopher Jürgen Habermas argued that, in the positivistic and technocratic environment of contemporary society, the emancipatory force of knowledge can only be regained by actively recovering the ‘forgotten experience of reflection’ ([1], p. 9). Without reflection, Habermas argued, human beings will become the objects and targets (rather than the autonomous subjects) of technocratic knowledge production systems, so that ultimately, even cognitive labour (i.e. scientific research) will be transmitted to robotics and smart machines. Indeed, he anticipated that, notably on the mental level, human beings will become increasingly dependent on intelligent contrivances. And eventually, humans may become ‘living accessories’ in a planetary machine park. Yet, unfortunately, Habermas argued, in the contemporary world, reflection is increasingly discarded as irrelevant.

In this paper, we will argue that, in the current situation, Habermas’ diagnostics of the present seem more pertinent than ever. The question to be addressed is how critical reflection can be maintained or restored in a contemporary setting. We will argue that, compared to more traditional types of reflection (‘desk-research’ or ‘armchair philosophy’), critical reflection requires a deliberative ambiance, a dialectical dialogue, a process of mutual learning, actively organised in various settings more or less simultaneously, in short, a process of deliberative and distributed reflection. And this especially applies, we will argue, to critical reflection concerning a specific subset of technologies which are actually oriented towards the human brain. By this, we mean particular substances and devices that are developed for the purpose of optimising cognitive performance, in other words, neuro-enhancement. Whilst new nootropic substances promise to allow individuals to become the managers of their moods, sleep behaviour and levels of attention, non-invasive brain stimulation (NIBS) devices such as tDCS, tACS, TMS and tFUS^1^ promise to help individuals to achieve everyday goals such as paying attention, relaxation, improving one’s gaming skills or learning to play a musical instrument faster. To ensure that these developments will function as technologies of freedom, rather than as technologies of control, a process of critical, deliberative and distributed reflection is required, which should be hands-on and well-informed, rather than academic. Whereas, academic discourse has been dominated by the debate between ‘transhumanists’ and ‘bioconservatives’ concerning the question whether human nature should or should not be drastically rebooted, often building on rather far-fetched thought experiments, an upstream process of distributed reflection, involving multiple voices, may broaden the spectrum and add some realism to deliberations on these issues (cf. [2]).

To achieve our goal, we participated in a European 7^th^ Framework Programme (FP7) project called NERRI (neuro-enhancement responsible research and innovation).^2^ In the context of this project, more than sixty mutual learning exercises (MLEs) were organised in 11 European countries [3]. In this paper, we analyse these exercises primarily from a methodological and conceptual point of view. To what extent can MLEs indeed create an ambiance for critical, deliberative and distributed reflection? What are the key concepts, tools and methods that can help us to realise this objective?

The design of our paper is as follows. First of all, we will briefly describe the ways in which the concept of reflection has been operationalised in the context of European research programmes, notably the 7th Framework Programme and the H2020 programme launched by the European Commission. We will especially focus on the ELSA concept (which stands for ethical, legal and social aspects of emerging science and technology) and the RRI concept (which stands for responsible research and innovation). Subsequently, we will explain the concept of a mutual learning exercise as a setting meant to foster deliberative and distributed reflection. Notably, attention will be given to key aspects such as ambiance and expertise, the role of ‘genres of the imagination’ and the profiles of various ‘subcultures of debate’. Subsequently, the MLE concept will be elucidated with the help of three case studies taken from the NERRI experience. Finally, we will discuss the added value of a mutual learning approach as a way to operationalise the ‘forgotten experience’ of reflection.

### Operationalising Reflection: from ELSA to RRI

The aim to initiate public deliberation concerning emerging technologies has been an important feature of European research policies since the 1990s. In the context of the 4th EU Framework Programme (1994–1998),^3^ a new concept was launched, namely ELSA: research and public engagement concerning the ethical, legal and social aspects of emerging sciences and technologies [4, 5]. This label was subsequently adopted by several other funding initiatives [6]. Especially, in the period of 2002–2012, a broad range of ELSA studies were conducted in Europe. Interdisciplinarity and proximity to scientific research were important features of ELSA activities, which usually took the form of flanking projects, embedded within larger scientific research programmes and developed in big science areas such as genomics and nanotechnology. These ELSA activities were both applauded and criticised, however. Some commentators considered ELSA as too critical (focussing primarily on possible downsides and risks of new technologies), whilst others considered it as too pro-science (or at least as pre-formatted by the scientific research programmes they intended to study, focusing on only a limited set of issues, often in the form of a case study research).

In the context of the 7th Framework Programme of the European Commission (2007–2013), a new label was adopted, namely, responsible research and innovation (RRI; [7–9]). According to René von Schomberg [10, 11]), one of the advocates of this approach, RRI is ‘a transparent, interactive process by which societal actors and innovators become mutually responsive to each other with a view to the (ethical) acceptability, sustainability and societal desirability of the innovation process and its marketable products (in order to allow a proper embedding of scientific and technological advances in our society)’ (Schomberg [11], p. 9; [10], p. 19). At first glance, this definition does not add that much to the original ELSA concept, where interaction between societal actors and innovators was also a key component. Nonetheless, on closer inspection, some differences between RRI and ELSA can be discerned as well. In the context of RRI, for instance, ethics is primarily seen as a ‘stimulus’ for science and technology (Schomberg [12]). Another important difference, according to protagonists such as Von Schomberg, is that RRI is a more ‘positive’ concept, aiming to encourage innovation, rather than retarding it by dwelling on social concerns. Furthermore, much more attention is given to the macro-economic impacts. RRI aims to contribute to the ambition of the European Union ‘to ensure that research and innovative ideas can be turned into products and services that create jobs and prosperity, as well as help preserve the environment and meet the societal needs of Europe and the world’ ([10]; cf. [9], p. 15). RRI aims, one could say, to prepare the society for innovation and to create a more responsible innovation for and with society. Unlike ELSA, in short, RRI first and foremost focuses on enhancing the economic development. The inclusion of ethics at a relatively early stage of the innovation process, it is argued, will lead to less contestation further down the line.

As a final distinctive feature, one could mention that, although ELSA already tended to display a relatively strong hands-on profile, in the case of RRI, the tendency towards pragmatism is even more outspoken. RRI documents reflect an explicit interest in the development of concrete tools for furthering responsible innovation in academia, industry and policy. One of the RRI projects funded under the FP7 call, for instance, is explicitly entitled RRI Tools. This project aims to ‘foster responsible research and innovation with and for society’.^4^ According to RRI Tools, RRI is a ‘dynamic, iterative process by which all stakeholders involved in the research and innovation practice become mutually responsive and share responsibility regarding both the outcomes and process requirements’. It aims to create ‘a society in which research and innovation practices strive towards sustainable, ethically acceptable, and socially desirable outcomes’ and it ‘does so in such a way that the responsibility for our future is shared by all people and institutions affected by and involved in research and innovation practices’ (RRI [13]).

This brief overview indicates that, although the definition of RRI is still relatively fluid, the concept seems to be evolving into a governance tool, aimed to encourage economic innovation by involving various (potential) stakeholders into the innovation process, thereby making them co-responsible for the process and its impacts. Hence, we would argue, RRI may be understood as a tool for research policy makers to engage society into policy decisions concerning emerging science and technology.

Yet, this tendency towards governance and valorisation is counteracted by various initiatives to ‘broaden’ the RRI concept [14] and to open it up to multiple forms of societal dynamics. The NERRI project, we will argue, funded by the 7th Framework Programme, is an example of this latter trend. Here, the RRI concept was operationalised in such a way that it became a mutual learning endeavour. The realisation of such an ideal, however, is bound to encounter serious challenges in practice [15–17]. First of all, although various approaches are being developed, ranging from constructive technology assessment (CTA: [18, 19]) up to the use of techno-moral vignettes for exploring future scenarios [20], this type of activity has not yet evolved into an established routine. Challenges include the fact that many of these newly emerging technologies are still fairly esoteric (so that their future impacts are indeterminate as yet and the precise agenda for deliberations is far from clear). Also, there is the possibility that such public engagement activities may be (perceived as) ‘symbolical’: creating a semblance of legitimacy, whilst the actual influence of participants on the developments at hand is limited. Therefore, this paper aims to contribute to an ongoing debate on how to optimally mobilise and involve stakeholders’ views, both conceptually and practically.

### RRI in Practice: Mutual Learning Exercises

As was already indicated, in the context of NERRI, the focus has been on mutual learning and upstream public engagement rather than on socio-economic impacts. There was hardly any involvement from industry in the project, for instance, although interviews with scientific experts as technology producers were part of the preparatory (reconnaissance) stage. In a recent NERRI report, the aim of RRI is described as follows: ‘The RRI approach aims to make new scientific and technological developments more transparent, interactive and responsive, so that acceptability, sustainability and social desirability of future applications can be proactively strengthened’ ([3], p. 9). Research and innovation are regarded as responsible insofar as they have the capacity ‘to adapt its direction in response to societal concerns, needs and values’. RRI is not seen as a specific method, but rather as “a basic attitude, an ethos if you like, seeing societal stakeholders not as ‘consumers’ of knowledge, but as sources of information and inspiration” (idem). By sharing preliminary analyses and assessments, research can be made ‘more relevant and socially robust’. RRI means that researchers and other producers of knowledge and technology see themselves as active participants in innovation and public debate. Promoting proximity of science, policy and social debate is of key importance ([3], p. 9). In other words, one might argue that NERRI is as much ELSA as it is RRI. The emphasis is more on promoting mutual learning than on boosting innovation.

Mutual learning exercises (MLEs) aim to bring together various groups of stakeholders (researchers, potential users, intermediaries, professionals, students, media, broader publics) to facilitate an interactive learning process through mutual exposure of views and experiences, expectations and concerns. The idea is that, in contrast with more traditional forms of deliberation (such as lectures, panel discussions or question-and-answer sessions before a relatively large audience), innovative methods must be employed to encourage in-depth dialogues. An MLE, one could argue, aims to function as a contemporary version of the ancient Socratic agora, providing a stage where multiple (and sometimes unexpected) perspectives are mutually exposed to one another, in order to move beyond traditional ‘experts vs. lay audience’ forms of exchange, thereby allowing participants to mutually probe and question each other’s views. The 60 MLEs represented a key element of the NERRI project and the aim of NERRI not only was to initiate a public debate on neuro-enhancement but also to explore and evaluate new formats for debate and interaction, so as to broaden the range of stakeholders/participants involved. Basically, an MLE addresses questions such as how will emerging neuro-technologies affect everyday lives of various segments of society? What are the anxieties and hopes, the expectations and concerns that are invoked and involved? These types of questions, anticipatory and open-ended, were addressed through deliberative and distributed forms of reflection. The goal was neither to reach consensus nor to further societal embedding, but rather to explore, articulate and open-up the issues at stake. An MLE aims to provide a deliberative laboratory where various hypothetical scenarios can be explored, developed and tested. By mutually sharing preliminary analyses and assessments, the deliberative process can be made more relevant, informed and socially robust.

### Distributed Expertise

By implication, the MLE concept entails a specific understanding of the role of expertise in the deliberative process. The idea is, first of all, that ‘all participants are experts’ in the sense of representing important views and experiences concerning the impact of technology on the life-world. In other words, expertise has become ubiquitous ([21], p. 15). Rather than taking a presumed knowledge deficit as point of departure, different forms of knowledge are distinguished and acknowledged as relevant for the deliberative endeavour. Subsequently, besides the fact that we are all experts to some extent, we all suffer from various knowledge deficits as well, also in the sense that the future is open and indeterminate and it is difficult to predict how technologies will evolve and how the life-world will be affected, in view of the messiness of the real world, outside techno-scientific laboratories. We are dealing with complex innovation processes, with technologies pervading the life-world, whilst they themselves will be affected by the way they are put to use. Whilst emerging technologies will affect and infect social culture, they will be infected by social and cultural dynamics as well. While coming to terms with new technologies, in a process of ‘vulnerability coping’ [22], future users will inevitably co-shape the development of these technologies. Laboratories are simplified environments, protected from intrusion and complications (‘noise’). Science tries to suspend or set aside the real messiness of the world in order to understand the noumenal dimensions of nature [23], but eventually, the real, phenomenal world as such cannot be understood or reached without acknowledging the complexity, the ‘mess’. Therefore, in order to extrapolate laboratory knowledge to real-life conditions and real-world practices, mutual learning is indispensable. The objective of MLEs is not to popularise or legitimise new forms of knowledge and technology, but rather to emphasise the complexities of the social world and to improve the societal embedding of these technologies by actively involving future users in the development process at a relatively early stage and in a co-constructive, upstream way.

Thus, rather than denying the expertise of academic specialists such as neuroscientists, multiple forms of relevant expertise are mobilised, taken into account and given the floor. In other words, the focus is not only on the expertise of the expert but also (or even more so) on the knowledge gaps, the uncertainties, the controversies, the unknowns, the blind spots, the epistemic vulnerabilities and the open future. Rather than disavowing expertise, the idea is that also for the expert, mutual learning is a more interesting and enriching experience than mere ‘popularisation’ of research, whilst other participants tend to learn more when they can enter into an active dialogue, compared to more passive forms of public involvement. Scientists work on multiple ‘floors’ (besides laboratory settings or academic podiums) where they may encounter various types of actors (journalists, funders, policy makers, entrepreneurs, etc.) and MLEs offer an additional floor [24]: an ambiance which fosters deliberation and reflection, not only for citizens (potential future users) but also for scientists themselves. Thus, MLEs are meant to provide a podium for actively exploring issues such as hands-on learning and the role of expertise. There is a shift of focus from what experts already know to the unknown (but nonetheless explorable) future. A special role may be played by genres of the imagination (art, novels, cinema and the like), inciting us and helping us to systematically explore multiple future scenarios.

### Cultures of Debate

When analysing the results of the more than 60 MLEs organised by NERRI, the picture emerges that the neuro-enhancement debate actually unfolds on different levels or stages, involving different ‘subcultures’ of debate. Overall, our experience has been that at least three deliberative subcultures can be distinguished ([3], p. 12 ff.; p. 25).

The first subculture consists of the so-called early adopters, eager to experiment with new nootropic substances or new types of non-invasive devices, in order to enhance, expand or boost the mind, for instance in the context of brain hackathon events and similar lab-like ambiances.

Next, there are audiences who are interested in the neuro-enhancement topic in principle, but who remain nonetheless rather sceptical about the possible benefits of these new substances and devices, compared to the possible risks, side effects and collateral damage they may entail.

Finally, there is a group of more cautious or even suspicious citizens who tend to critically oppose neuro-enhancement innovations, not so much because of specific health risks involved, but rather because of the socio-cultural implications they entail. These innovations symbolise a society they do not want, reflecting for instance a neo-liberal atmosphere of cognitive capitalism, fostering socio-economic competition among individuals, who (according to these voices) are framed as brain managers or cognitive entrepreneurs.

This triad division is still fairly general and may be differentiated further. MacNaghten et al. [25] for instance use five narratives to elaborate reluctance or even suspicion concerning emerging technologies (using nanotechnologies as their case study), namely (1) ‘be careful what you wish for’ (voicing reluctance concerning the seductive temptations of emerging technologies); (2) ‘opening Pandora’s box’ (stressing uncertainties and potential catastrophic implications of meddling with things that should be left alone; (3) ‘messing with nature’ (emphasising disruptive impacts of tampering with nature); (4) ‘kept in the dark’ (articulating a sense of powerlessness, a (perceived) inability to have any impact in these technological developments); and (5) ‘the rich get richer and the poor get poorer’ (fearing increase of injustice and inequality). In organising MLEs, it is important to keep such distinctive deliberative subcultures in mind. Developments which may seem highly futuristic or science-fictional to many may already be part of everyday life for others. The latter notably applies to avant-garde subcultures of youngsters who are keen on exploring options for enhancing oneself or embracing the technology dense, robotic future.

One of the problems involved in distinctions between various deliberative subcultures is a semantic one, namely, the fact that the labels employed may convey pejorative or stereotypical associations. The concept of ‘early adopter’, for instance, was introduced by Rogers [26] in the context of his theory of diffusion: a theory which builds on the normative idea that ‘early adoption’ = good, whereas ‘late’ adoption or even ‘resistance’ = bad. From a mutual learning perspective, such normative framings of the various positions in the debate are questionable, to put it mildly. Openness to novel techniques is not necessarily good (it may rather involve a naïve underestimation of the collateral damage of certain drugs or certain brain-stimulating devices, for instance), whilst scepticism or conservatism are not necessarily bad (notably in view of the various hypes at work in the current innovation arena, for instance). Rather, the mutual learning model entails that all types of responses are to be taken equally seriously in the debate, and multiple factors may co-determine attitudes towards innovations in the neuro-enhancement field.

We will now outline three other important factors that may either stimulate or thwart the deliberative reflection process which the MLE format aims to promote.

### The Role of Ambiance

For conducting MLEs, location or, more generally, ambiance proves to be an important element (cf. [3], p. 28). Whereas, more traditional debates (lectures by, or panel discussions among, experts before a relatively large audience, for example) tend to be organised in more or less traditional settings (such as lecture halls at universities or debating centres), mutual learning exercises are preferably organised in more ‘thought-provoking’ environments such as brain labs, future labs, design labs, art labs, science cafés and science museums; settings that are optimally poised to serve as imaginative laboratories and to encourage societal ‘experiments’ (imaginative explorations of the future), or to demonstrate, in a hands-on fashion, novel various devices and ideas (cf. [27]). In science cafés, for instance, space can be consciously employed to create multiple perspectives within an informal setting (participants sitting in groups, on bar stools, standing at the bar, etc.), whilst the presence of enhancement devices (in science museums, for instance) may add a hands-on, explorative supplement to the deliberative debate.

Several NERRI events were conducted in science museums as the spaces specialised in exploring the interface of science, technology, society and art. One event was organised in a planetarium, for instance,^5^ placing participants literally in the dark, contemplating enhancement underneath a celestial dome and respecting two rules: the silence rule (expecting participants to wait for a moment of silence before speaking) and the continuity rule (requesting that every intervention should be related to the preceding one). At a certain point, the lights were turned on once again so that participants could openly discuss the knowledge that was collaboratively (and more or less anonymously) created.

Another NERRI event, dedicated to exploring the question of digitally ‘dissecting’ and subsequently ‘improving’ the ‘criminal’ brain, was organised in an exposition room located in a historical seventeenth-century building named Dolhuys, which literally means ‘asylum for the insane’.^6^ The context itself already raised the question how to frame current brain enhancement debates against the backdrop of centuries of intervention, (mis-)treatment and (mis-)measurement.

One of the brain hackathons in which NERRI partners participated was organised in a fifteenth-century building where (during the early modern period) an anatomical theatre had been located, thereby connecting old and new ‘enactments’ of brain research. Such settings add a sense of history, a temporal horizon to the debate and often provide room not only for deliberations but also for try-outs and demonstrations, whilst the atmosphere facilitates comparisons between past, present and future, seeing emerging trends against a broader cultural and epistemic backdrop. Besides arguments and words, also visualisations, colours, space, architecture, sound (acoustic ambiance or soundscape) and other sensory/spatial components contribute to and influence the debate.

Traditional settings of public debates tend to be mono-logical or, at best, elliptic, with two focal points (‘centres’) as it were, for instance a neuroscientist and a bioethicist, addressing a largely passive, receptive audience, but NERRI aimed to provide testbeds for developing and probing innovative formats whose common objective has been to make the debate more horizontal and decentralised, topologically speaking.

### Employing Creative Tools

A number of deliberative encounters organised by NERRI involved the use of games and special assignments, to be conducted in small groups. Several MLEs, for instance, used Play Decide, a conversation game involving between four and eight participants and a moderator, sitting around a table. Each kit contains a set of cards describing neuro-enhancement cases and raising legal and ethical issues. Participants share, discus and defend their viewpoints, until a certain level of agreement is reached. A similar exercise involved a 90-min ‘discussion game’ using various types of cards with information on technologies, prevalence and health issues: stories told from different angles and introducing various ethical and social questions.

Another example of an innovative format is the so-called World Café.^7^ This format usually involves 20-min rounds of conversations in small groups seated around a table, prompted by specific questions, taking notes with the help of markers on large sheets of paper. After 20 min, group members move to a different table. Subsequently, participants are invited to share insights and deliberative results with the other teams. These results are reflected visually in a variety of ways, using white boards, graphics, posters and the like. Again, another example was a seminar in which participants were invited to write initial statements concerning techniques such as deep brain stimulation (DBS) and tDCS on cards, after which the group ranked these statements in order of importance. The most important statements subsequently served as input for a convergence exercise, during which participants changed tables between rounds.

Such innovative deliberative tools often involve the use of multiple media. Thus, besides words, arguments and architecture (ambiance, topology), visualisation is an important dimension as well. For instance, creating specific props can be useful, such as the mock NE kit that was used during some NERRI MLEs, involving flasks with candies and electronic equipment, but also labels conveying basic information on enhancement drugs and devices, its effects, warnings, contexts of use and availability of funding to prompt the debate. And the World Café already mentioned above involved not only a professional video recording of the event but also a professional cartoon artist who produced a visual impression on site during the day (a fragment is displayed on the left). Her drawing provided an artistic reflection in the introductory talks and the workshop discussions, based on observations, associations and quotes. The result was discussed by participants at the end of the meeting.

During another MLE, an artist commented on his personal experience with enhancement. And in again another MLE, participants witnessed artistic performances with EEG headsets, mind-ball game and other devices, trying it out on themselves. Still, another NERRI MLE involved a playful exploration of memory skills, demonstrating the potential of training your memory with the help of various artistic mnemonic devices. The conclusion was that art may provide inspirational tools for boosting public awareness concerning the impact of on the one hand (visual, auditory or motor) handicaps or impairments and on the other hand, enhancements devices meant to optimise cognitive performance. In a hands-on way, and from a first-person perspective, both restricted and enhanced forms of mental functionality could be experienced and compared with normalcy. In other words, via imaginative tools, verbal deliberation may be complemented by thinking with your hands, with your sense organs, actively probing and trying out new options for mobility and perception.

### Genres of the Imagination

Building on the experiences of others [28, 29],^8^ we explicitly explored how genres of the imagination (such as novels, plays, movies, poems and art-works) can be employed as imaginative laboratories of deliberative reflection, as windows into science and as podiums where future scenarios involving emerging technologies may be explored and assessed in a more or less experimental fashion.

The idea that drama and theatre can play an inspirational role in deliberations on the future societal implications of science and technology, for instance, is wide-spread [30, 31]. Usually, this involves the performance of a play by professional or amateur actors before a live audience of, say, students or lay citizens, as happened for example during one particular MLE event where three scenarios were performed by a university theatre group presenting risks and various options, side effects and future perspectives of DBS. In another MLE (which will be discussed in more detail below), science students were invited to write and perform a play of their own, and the winning team was asked to perform it during a science café.

Several MLEs made use of cinema as a tool for opening up the field and for stimulating the debate. Two MLEs used the movie *Limitless*, for instance, released in 2011 and involving a writer suffering from a writer’s block, until he tries a pharmaceutical brain enhancer. Gradually, the collateral damage of the experimental designer drug becomes increasingly evident (cf. [32]). In five NERRI MLE events, the documentary *Fixed* (an imaginative, multiple-voice portrayal of possible enhancement scenarios) was used, on one occasion in combination with a live testimony by a cochlear transplant patient. Such materials made the topic concrete and tangible for the audience. In evaluations, participants indicated that everyday consequences become more evident and memorable than those in the case of, say, academic discussions between experts. *Fixed* presents enhancement from multiple perspectives, each connected with a particular face and story line, which makes it particularly valuable for this purpose, allowing participants to identify with concrete individuals and their specific viewpoints, whilst at the same time, making it clear that a particular perspective is not the only one possible. This combination of a counter-balanced, but still touching and involving presentation, staging various ‘voices’ as it were, would have been difficult to reach through other means. During evaluations, attendants indicated that *Fixed* addresses controversial but fascinating issues, thereby providing plenty of food for thought and giving rise to engaged discussions ([3], p. 35–36).

Rather than merely ‘illustrating’ moral dilemmas, moreover, documentaries, movies and theatre plays may present a stage for exploring, probing and reflecting on aspects that are more difficult to address with the help of usual methods (lectures, group discussions, panels and the like). Such performances may succeed in giving a face to a topic, and allow for emotional identification and reflective distancing at the same time. They may serve as windows into everyday life situations where neuro-enhancement may have an impact. More traditional and abstract methods (such as lectures or group discussions) appear to have a less memorable impact on participants (cf. [30]).

### MLE Case Studies

To further explore the MLE concept, three examples of MLEs will now be presented in more detail. In the final section, the added value of such exercises will be outlined.

#### Case Study 1: Super Me

Case study 1 is an MLE organised in June 2014 by NERRI partner Experimentarium, a science museum/science activity centre located in Copenhagen (Denmark).^9^ Experimentarium’s aim is to encourage interest in science and technology especially among children and youngsters.^10^ The MLE aimed to challenge the way people think about human enhancement and to spur a debate on where the line for human enhancement should be drawn. To do this, a role-play game was developed entitled Super Me. Visitors were invited to participate in this game and to subsequently join the debate on how far we should go in optimising the brain. Role playing was expected to involve participants more actively in the discussion, allowing the organisers to present neuro-scientific developments in such a way that it became possible for young audiences to explicitly relate to them.^11^

The Super Me event staged a fictitious conference that was taking place in the future, exactly 50 years from now. The world would be in chaos because citizens of all nations were using brain optimisers. The aim of the game was to try and reach global agreement to regulate the enhancement arena. Visitors who joined the event were invited to enter into a dialogue with other role-game players and to take a position vis-a-vis six ethical dilemmas. Furthermore, high school students who had been studying different areas of neuro-enhancement were invited to present their findings whilst the other attendants could pose questions to these students, for example in order to gain a more thorough understanding of a specific brain technology.

Thus, visitors were invited to become part of a scenario and to experience the complexities of a specific topic in a situation where decisions had to be made. The idea was that live action role play would strengthen the learning process and make the issues involved more understandable, concrete and meaningful. Human enhancement is focussed on the individual self, but at the same time embedded in social relationships and cultural trends, and participants were invited to actively relate to these broader dimensions. The collective experience of setting up a role-game allowed the participant to use their imagination in order to connect neuro-enhancement developments to daily life, thus making the dilemmas involved more striking and tangible.

In terms of results, qualitative information concerning evaluations and experiences of visitors was collected via exit polls, snap-shot interviews and an online questionnaire. It indicated that the role play allowed a large number of visitors (circa 1200 in 2 days) to become actively involved in the debate and develop their views. Whereas, traditional setups such as lectures or panel debates tend to focus on the present and involve visitors primarily as audience, the role players acted as visitors from the future as it were, broadening the temporal horizon of the debate and allowing participants to articulate and develop their views through dialogue. A broad range of topics could thus be addressed (from the role of creative use by consumers in technology development via normalcy and competitiveness to good governance, equity and access). Many visitors indicated, moreover, that they saw enhancement as something that was already happening. Overall, participants confirmed that they not only increased their knowledge about the topic, but were also encouraged to explicitly consider their own opinion on the matter. Role-playing games are particularly an option when audiences are invited to discuss innovations which are potentially controversial, but at the same time relatively early stage. In order for the role-playing game to work and mutual learning to take place, however, some conditions have to be met. First of all, for visitors to successfully participate in such a learning event, a basic understanding of issues and current options is a prerequisite. But, also the role players themselves should be knowledgeable about the topic and trained to effectively engage with visitors. In this way, role playing allows participants to become part of the on-going, distributed deliberation concerning neuro-enhancement. But before assessing the added value of MLEs such as Super Me more elaborately, two other case studies will be briefly presented.

#### Case Study 2: SuperMI

Case study 2 is an MLE organised by the Universitat Pompeu Fabra (UPF), Barcelona (Spain), entitled SuperMI and aimed to provide an open platform for citizens to discuss neuro-enhancement technologies, notably technologies developed to optimise the cognitive capacities of healthy people. In terms of ambiance, the debate was organised in CosmoCaixa, a well-known science museum in Barcelona, in November 2014. The session had the format of a science café with participants seated around round tables whilst beverages and pastries were offered. The atmosphere was decidedly informal, and speakers (coming from various positions within the enhancement field) were encouraged to involve the audience, so that the whole session (facilitated by moderators) evolved into a dialogue. The topics discussed focused on neuro-ethics and the social and economic impacts of introducing neuro-enhancement in everyday life. Active involvement of the audience was also strengthened with the help of a voting system, as a preparatory exercise culminating in a final open debate among all participants. The interactive voting system^12^ used remote controls (user-friendly to all age groups) which allowed participants to respond to various options presented to them. These responses were publicly displayed and commented on the spot, to further enhance the debate.

The majority of the audience approved the use of enhancement substances and technologies in healthy people to prevent or postpone age-related cognitive problems (such as memory loss), but tended to be more negative with regard to recreational enhancement tools.^13^ Evaluation questionnaires collected immediately after the event indicated that participants, besides being intrigued by the topics discussed, appreciated both the real dialogues during the session and the real-time voting system. The voting system provided a mirror to determine whether individual views are mainstream or exceptional. Thus, this combination of tools allowed participants to position themselves in the debate.

#### Case Study 3. Drama in a Science Café

Case study 3 is a science café organised by NERRI partner Radboud University Nijmegen. As a preparatory exercise, we invited 22 science students who participated in a philosophy course on neuro-enhancement to write and perform a play of their own addressing the social and ethical dilemmas involved. The plays were presented during one of the sessions of the course, and the winning team was invited to perform their play on stage during the NERRI Science Café, before a live audience of about 130 visitors ([3, 30], p. 34). Our question was: what will students gain from such an experience? We opted for drama because a basic affinity can be discerned between drama and experimental research. Whilst experimentation itself can be regarded as a dramatic form of research (often involving enactments in front of an audience of students, colleagues, or even journalists etc.), drama may function as an ‘ethical laboratory’ to explore and critically reflect on possible ethical and societal implications, now or in the near future ([3, 30], p. 34).

To assess students’ learning experiences, three questionnaires were developed and distributed, before, during and after the course. The winning team enacted a competition between a natural and an enhanced physician (equipped with a mnemonic device). The vicissitudes of both competitors were visible on the podium, for instance because, whilst one role was being performed, the other was temporarily frozen and vice versa, which strengthened the element of contrast: the awareness that there are multiple options. The dramatic performance proved rather effective and contributed to the liveliness of the ensuing debate; for instance, because several participants from the audience used the play to support their views. At a certain point, one of the visitors asked the rest of the audience to choose between the two protagonists: would they want to be treated by a natural or an enhanced physician? In response to this question, and somewhat surprisingly perhaps, about half of the audience indicated a preference for being treated by the enhanced expert, who was less sensitive to emotional and social aspects of illness, but had more factual knowledge available due to the mnemonic device. This stimulated the discussion as participants began to discuss and question the interpretations and preferences of others. In their assessment of impact and results, however, the organisers focussed on the learning experience of the students (*N* = 17). They measured self-reported learning outcomes using a five-level Likert scale, with responses ranging from 1 (totally disagree) up to 5 (totally agree). In response to the item ‘The theatre assignment helped me to gain insight in how emerging technologies could influence daily life’, the mean score was 4.24 (with 92% of the participants scoring 4 or 5). And in response to the item ‘The theatre assignment helped me to gain insight in other people’s opinions and arguments’, the mean score was again 4.24 with 88% of the participants scoring 4 or 5 ([30], p. 885). Moreover, as one of the students commented: ‘That we succeeded in stimulating thoughts on this issue was proven by the fact that a lot of the topics in the discussion following the play were directly linked to the issues we touched upon in our act, culminating in a debate with the audience on whether people would rather be treated by the rational and enhanced or by an empathic but non-enhanced doctor; a vote split the audience roughly in half’ (p. 886).

## Discussion

Building on the MLE concept, all three case studies discussed above adopted creative ways to literally involve the audience: allowing audiences to become an active actor in an experimental deliberative performance. In case study 1, the participants communicated with future generations about real-life experiences; in case study 2, the opinions of participants were projected real-time on stage, so that participants could actively relate to the evolving agreement or disagreement; and in case study 3, both the students and their audience became involved in enacting the moral dilemmas of enhancement technologies. In all three cases, the active involvement of the audience was used to stimulate and deepen the debate. The experimental performances became a kind of demonstration or public experiment, aimed at exploring future scenarios in outline. That is, these experimental tools not only affected the experiences of the participants who were directly involved in the performances, but also stimulated debates and learning processes among other sections of the audience. The question now is to what extent these case studies, in combination with the considerations concerning ambiance, expertise, creative tools and imagination presented above, allow us to further develop the MLE concept.

The question ‘what is (the general profile of) an MLE’ has already been addressed above. An MLE, we argued, aims to bring together various groups of stakeholders (researchers, potential users, intermediaries, professionals, students, media, etc.) to facilitate an interactive learning process through mutual exposure of views and experiences, expectations and concerns. Innovative methods (such as drama or games or voting systems) are employed to encourage active participation, allowing the MLE to become a contemporary version of the Socratic agora, providing a stage where multiple (and sometimes unexpected) voices and perspectives mutually challenge each other: the deliberative version of the Bakhtin’s concept of ‘heteroglossia’ [33]. An MLE aims to provide a deliberative and distributed form of reflection. Instead of allowing a limited number of ‘reflection professionals’ to analyse current developments in the neuro-enhancement arena, reflection ideally becomes a joint and mutual learning endeavour. Thus, a dialogue on neuro-enhancement ensues which exceeds traditional experts-versus-lay audience roles. This does not mean that the expertise of the scientific expert is disavowed; rather it is mobilised in more interactive ways, which entails a learning experience for the scientific experts involved as well. In the course of this joint endeavour, a living laboratory is created in which scientific and societal experts jointly develop plausible scenarios and critical assessments of controversial topics such as neuro-enhancement.

Compared to more traditional deliberative settings such as lectures or panel debates, MLEs require more explicit planning, first and foremost during the preparatory stage. And this not only involves the design of the meeting and the development of innovative tools, but also aspects such as diversity and inclusion (the aim to reach out to broader sections of civil society) as well as responsiveness (the debate must be framed and phrased in such a way that it is responsive to current developments, not only in research laboratories, but also in society at large). This implies that MLEs are time-consuming and cannot be regarded as mere add-ons to techno-scientific projects. As reflected in evaluations of MLE events, audiences appreciate active participation as it allows them to position themselves more explicitly in the debate. Whereas in this paper the focus was on conceptual and pragmatic considerations for organising such events, it would certainly be worthwhile to determine more precisely (and also more quantitatively) the learning effects entailed. Although in case studies 1 and 2 exit polls and snap-shot interviews suggested that mutual learning actually resulted from these activities, scales such as the ones adopted in case study 3 allow organisers to test their expectations more explicitly, thus furthering the learning process of the organisers themselves. In other words, employing calibrated evaluation tools (explicitly involving the role of ambiance, imaginative tools, role play etc.) may enable organisers to determine learning impacts with more precision. Further progress in this direction is implied in taking the MLE concept seriously.

The MLE concept reflects and builds on the tendency towards pragmatism of the RRI approach. With the help of MLEs, reflection becomes a deliberative praxis. Instead of the traditional situation of academic experts providing policy advice, experts are invited to become involved in a deliberative process meant to encourage reflection not as an isolated phenomenon (pursued by expert committees etc.), but emerging in the context of a broader deliberative culture, as a contemporary version of what Hegel [34] once referred to as *Sittlichkeit*: the ethical life, the practical realisation and enactment of the reflexive ideal, facilitated by a supportive, consciously organised deliberative environment. Via their active involvement in imaginative, social laboratory settings, designed to explore and critically assess possible scenarios, citizens become the co-authors rather than the targets of technological change.

## Footnotes

1 These acronyms refer to transcranial direct current stimulation (tDCS); transcranial alternating current stimulation (tACS); transcranial magnetic stimulation (TMS); and transcranial focused ultrasound stimulation (tFUS).

2
http://www.nerri.eu/eng/home.aspx

3
https://ec.europa.eu/research/fp7/index_en.cfm

4
http://www.rri-tools.eu/; http://www.rri-tools.eu/about-rri

5
http://www.nerri.eu/eng/mutual-learning-exercises/mle-32-cosmological-caf%C3%A9.aspx [accessed March 29, 2016]; cf. [3], p. 29

6
http://www.nerri.eu/eng/mutual-learning-exercises/mle-55-haarlem-dolhuys.aspx [accessed at March 29, 2016].

7
http://www.nerri.eu/eng/mutual-learning-exercises.aspx

8 Fascinating examples concerning the use of genres of the imagination in up-stream assessment of emerging technologies are provided by *Biofaction* (http://www.biofaction.com/) and the *Critical Art Ensemble* (http://critical-art.net/), among others.

9
http://www.nerri.eu/eng/mutual-learning-exercises/mle-13-super-me.aspx [accessed March 29, 2016]

10
http://en.experimentarium.dk/

11
https://www.youtube.com/watch?v=Eioy_sIICiU

12
http://www.powervote.com/nl/

13
http://ccs.upf.edu/en/supermi-at-cosmocaixa-an-open-citizen-debate-to-talk-about-neuro-enhancement/
